# Comparison of culture-based methods for detecting *Campylobacter jejuni* and *Campylobacter hyointestinalis* in dairy cattle feces

**DOI:** 10.1128/spectrum.01475-25

**Published:** 2025-08-15

**Authors:** Krista Tuominen, Ingrid Hansson, Robert Söderlund, Stefan Bertilsson, Reza Belaghi, Lena-Mari Tamminen

**Affiliations:** 1 Department of Clinical Sciences, Swedish University of Agricultural Sciences 8095, Uppsala, Sweden; 2 Department of Animal Biosciences, Swedish University of Agricultural Sciences 8095, Uppsala, Sweden; 3 Department of Microbiology, Swedish Veterinary Agency 8094, Uppsala, Sweden; 4 Department of Aquatic Sciences and Assessment, Swedish University of Agricultural Sciences 8095, Uppsala, Sweden; 5 Department of Energy and Technology, Swedish University of Agricultural Sciences 8095, Uppsala, Sweden; Michigan State University, East Lansing, Michigan, USA

**Keywords:** animal reservoirs, culture methods, bacterial pathogens, zoonoses

## Abstract

*Campylobacter* is the most commonly reported cause of bacterial gastroenteritis in humans. Although cattle are recognized as a potential reservoir for several *Campylobacter* spp., most detection standards primarily target thermotolerant species, notably *Campylobacter jejuni* and *Campylobacter coli*, possibly underestimating the prevalence of others. This study evaluated the performance of different culture-based methods for detecting *Campylobacter* spp. in fecal samples collected rectally from dairy cows in a single commercial research herd across four time points. Six combinations of analyses were tested, involving either direct culture or enrichment broths (Preston and Bolton) paired with selective agar media (modified charcoal cefoperazone deoxycholate agar or Preston). Incubation was performed at 37°C under microaerobic conditions to support growth of non-thermotolerant species. Species identification was performed using matrix-assisted laser desorption/ionization time-of-flight mass spectrometry (MALDI-TOF MS). Only *C. jejuni* and *Campylobacter hyointestinalis* were detected. While *C. jejuni* prevalence declined over time, *C. hyointestinalis* remained consistently high throughout the study period (June–August 2024). The performance of tested detection methods varied significantly between species. Enrichment with Bolton broth increased the odds of detecting *C. hyointestinalis* by over 3,000-fold compared to the direct culture (odds ratio [OR] = 3,075; 95% confidence interval [CI]: 272–34,651). For *C. jejuni*, enrichment with Preston broth increased the odds of detection by more than eightfold (OR = 8.52, 95% CI: 3.16–22.9). Detection was primarily influenced by broth; selective agar had no independent effect. These findings emphasize the importance of method selection in the detection of *C. hyointestinalis* and *C. jejuni*, suggesting that *C. hyointestinalis* may be more prevalent in cattle than previously assumed.

**IMPORTANCE:**

*Campylobacter* bacteria commonly cause gastrointestinal illness in humans and are frequently found in animals such as cattle. Detecting these bacteria in animal samples is important for understanding their occurrence and potential relevance to food safety. Many commonly used laboratory methods focus on *Campylobacter* spp. that grow under specific conditions, which may limit the detection of other species. This study compared several culture-based methods for the isolation of *Campylobacter* spp. from fecal samples collected from dairy cattle. Species identification was subsequently performed using MALDI-TOF MS. The findings show that detection varied, depending on the culture method and the *Campylobacter* spp., highlighting the potential impact of method choice on surveillance outcomes.

## INTRODUCTION

*Campylobacter* is a microaerophilic gram-negative bacterium commonly found in the gastrointestinal tract of several animal species (1). In humans, *Campylobacter* is one of the most common causes of gastroenteritis, most often linked to thermotolerant *Campylobacter jejuni* and *Campylobacter coli* (2–4). Certain non-thermotolerant *Campylobacter* spp., such as *Campylobacter fetus*, *Campylobacter hyointestinalis*, *Campylobacter iguaniorum*, and *Campylobacter lanienae*, form a distinct phylogenetic clade within the genus and are predominantly found in ruminants and other grazing livestock (5). Although infections in humans are mostly sporadic and less frequent compared to those caused by *C. jejuni* and *C. coli*, these species can still cause disease, particularly in immunocompromised individuals (6–8).

Transmission of *Campylobacter* typically occurs through consuming contaminated food, particularly undercooked meat and meat products, or contaminated water and unpasteurized dairy products (9–13). Although contaminated poultry products have been recognized as the major source of human campylobacteriosis, cattle also represent an important reservoir for *Campylobacter* spp. (14–17).

Previous studies have reported a wide range of prevalence (0%–83%) of *Campylobacter* spp. in cattle (15, 18–22). The prevalence seems to be higher in calves than in adult cattle (15, 19). However, the results from different studies are often not directly comparable because of the variation in laboratory methods and study designs. For example, two French studies conducted in separate herds reported markedly different *Campylobacter* spp. prevalence, depending on the method used: 69.1% with enrichment in Preston broth and 16.5% with direct culture (15, 23). *C. jejuni* and *C. coli* are two of the most frequently reported *Campylobacter* spp. in scientific literature regarding cattle, with prevalence of 7%–98% and 0%–12.5%, respectively (15, 20, 24–26). While *C. hyointestinalis* has received less attention, some studies have reported prevalences from 15.3% to 34.0% (20, 24, 25).

Reliable methods for the detection of microorganisms are essential for monitoring and surveillance. Detection methods for *Campylobacter* include culture-based techniques and molecular approaches such as PCR (27, 28). Standards, such as the International Organization for Standardization (ISO) standard for *Campylobacter* (ISO 10272, parts 1 and 2), provide guidelines for detecting and enumerating *Campylobacter* in food and animal samples. However, these standards primarily target thermotolerant species such as *C. jejuni* and *C. coli* (28) and may fail to detect the non-thermotolerant *Campylobacter* such as *C. hyointestinalis* and *C. fetus* (29, 30). In fact, protocols using lower incubation temperatures (e.g., 37°C) have succeeded in detecting several non-thermotolerant *Campylobacter* spp. in meat and bovine feces (31, 32), which may be missed when using methods with higher incubation temperatures (e.g., 41.5°C).

Although established standards for the detection of thermotolerant *Campylobacter* spp. exist, methodological variations between studies may lead to underestimation of the true prevalence and diversity of *Campylobacter* spp. in different sources. Improving surveillance for non-thermotolerant species is therefore important to better understand their prevalence in the food chain and their potential public health impact.

This study aimed to compare the performance of several culture-based methods for detecting *Campylobacter* in fecal samples from dairy cattle. Additionally, by varying the incubation temperature and combining different media, this study sought to enhance our understanding of the diversity of *Campylobacter* spp. in cattle.

## MATERIALS AND METHODS

### Study population and design

Fecal samples were collected from 18 cows in the dairy barn of the Swedish Livestock Research Center (Uppsala, Sweden). Each cow was sampled four times in June–August 2024 at 3- to 5-week intervals.

The cows were selected based on the breed and lactation phase (Holstein breed and less than 100 days from calving at the first sampling date). The final selection was based on the heterogeneous representation of the total number of calvings and the somatic cell count before the first sampling date (Table 1). Cow 4 was removed from the herd after the first sampling due to a teat injury and was replaced with cow 9. Additionally, cow 8 was removed between the third and fourth sampling occasion due to severely reduced physical condition and blood in the feces.

**TABLE 1:** Description of the cows selected for the study and their parameters at each sampling occasion^*a*^

Cow	Sampling occasions	Age (mo)^*b*^	Lactations	Sampling occasion 1		Sampling occasion 2		Sampling occasion 3		Sampling occasion 4	
				DIM	SCC	DIM	SCC	DIM	SCC	DIM	SCC
1	1–4	72.5	5	9	50	36	18	57	18	92	4
2	1–4	68.0	4	68	92	95	16	116	13	151	12
3	1–4	62.7	4	29	17	56	13	77	68	112	61
4	1	54.3	3	24	4,081	–^*c*^	–^*c*^	–^*c*^	–^*c*^	–^*c*^	–^*c*^
5	1–4	42.6	2	67	108	94	29	115	15	150	149
6	1–4	41.1	2	7	90	34	9	55	9	90	59
7	1–4	40.9	2	48	32	75	13	96	34	131	33
8	1–3	40.1	2	47	9	74	29	95	4	130	–^*c*^
9	2–4	40.0	2	–^*c*^	–^*c*^	48	17	69	17	104	35
10	1–4	39.9	2	78	919	105	–^*d*^	126	–^*d*^	161	–^*d*^
11	1–4	38.5	2	26	950	53	–^*d*^	74	1,805	109	140
12	1–4	26.8	1	36	42	63	122	84	81	119	77
13	1–4	26.6	1	59	371	86	214	107	533	142	519
14	1–4	26.4	1	76	31	103	124	124	48	159	31
15	1–4	26.2	1	1	3,034	28	396	49	290	84	337
16	1–4	25.8	1	54	18	81	31	102	33	137	51
17	1–4	25.4	1	26	39	53	–^*e*^	74	39	109	56
18	1–4	24.5	1	17	39	44	20	65	56	100	28
19	1–4	24.3	1	18	122	45	253	66	204	101	136

^*a*^ DIM, days in milk; SCC, somatic cell count.

^*b*^ Age in months at the first sampling occasion.

^*c*^ The cow was not sampled at the corresponding time point.

^*d*^ Value missing due to an error in data transfer from the milking robot.

^*e*^ Value missing due to the cow being temporarily in the sick department.

### Sample collection

A large handful of feces was collected rectally from cows using a clean rectal glove (Eickemeyer KG, Tuttlingen, Germany) and less than one tablespoon of lubricant (VetGel; Albert Kerbl GmbH, Buchbach, Germany). Each sample was transferred to a clean 3 L plastic bag, which was emptied of excess air. The samples were transported chilled to the Swedish University of Agricultural Sciences. The analysis of all samples was started within 24 h of sample collection.

### Species detection and identification

**Fig 1:**
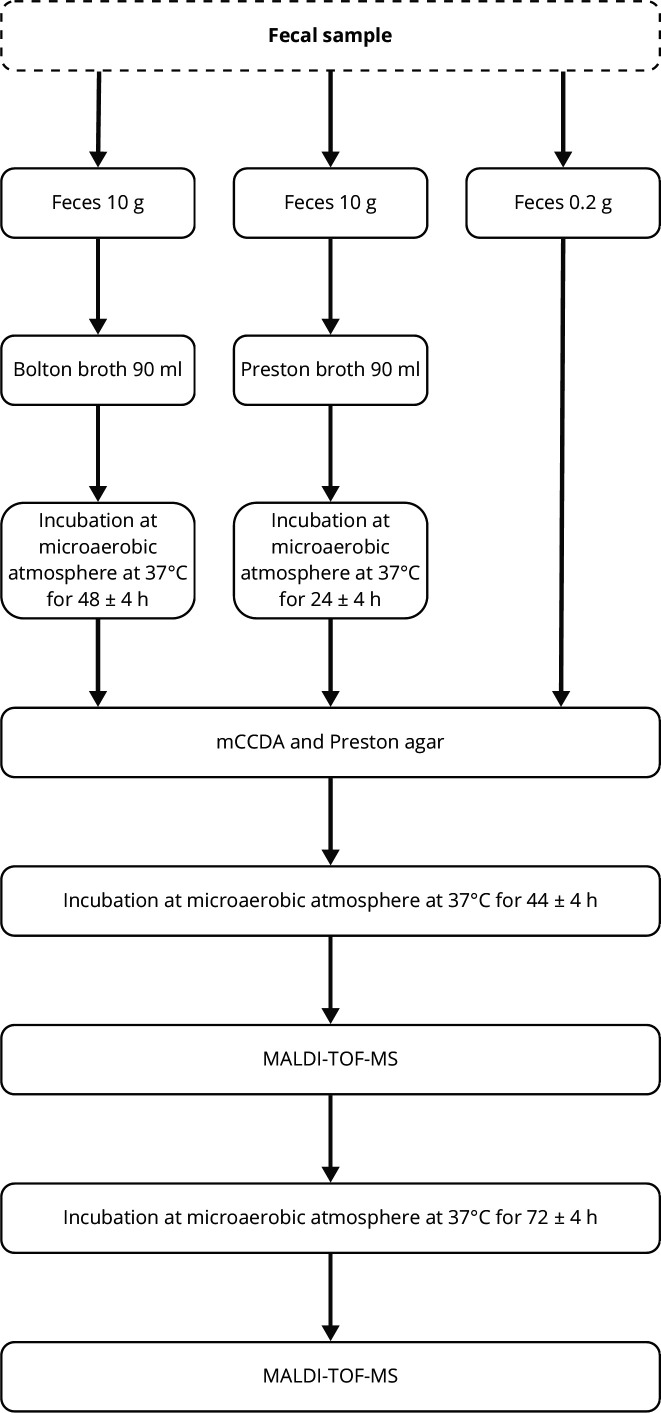
Diagram of the procedure used for detection and identification of *Campylobacter* spp. from cattle feces. The flowchart is modified from International Organization for Standardization 10272: part 1, annex A. MALDI-TOF MS, matrix-assisted laser desorption/ionization time-of-flight mass spectrometry.

A modified version of ISO 10272: part 1 (28), incorporating enrichment broth and agar plates, was used to detect and identify *Campylobacter* spp. (Fig. 1). In short, six culture-based method combinations were used: (i) direct culture on modified charcoal cefoperazone deoxycholate agar (mCCDA) agar; (ii) direct culture on Preston agar; (iii) Preston broth enrichment followed by culture on mCCDA agar; (iv) Preston broth enrichment followed by culture on Preston agar; (v) Bolton broth enrichment followed by culture on mCCDA agar; and (vi) Bolton broth enrichment followed by culture on Preston agar, as detailed below.

For enrichment, two separate 10 g portions of each fecal sample were added to 90 mL of Preston broth and 90 mL of Bolton broth (Oxoid, Basingstoke, England). Preston broth was incubated at 37°C for 24 ± 4 h, while Bolton broth was incubated at 37°C for 48 ± 4 h. These incubation times followed ISO 10272: part 1, but the temperature was reduced to 37°C ± 1°C to support the growth of non-thermotolerant *Campylobacter*.

Following enrichment, 10 µL of the cultures obtained from enrichment was spread on modified charcoal-cefoperazone-deoxycholate agar (mCCDA, Oxoid) and Preston agar (SVA, Uppsala, Sweden) and incubated at 37°C. All enrichment broths and samples were incubated in a microaerobic atmosphere using Campygen 2.5 L (Oxoid). Additionally, a loopful of fresh feces, about 0.2 g, was cultured directly onto mCCDA and Preston agars. After 44 ± 4 h of incubation on solid media, the agar plates were examined for bacterial growth. All colonies with a macroscopically distinct appearance from each other (based on differences in size, shape, color, and texture; total of zero to five colonies per plate) were identified with matrix-assisted laser desorption/ionization time-of-flight mass spectrometry (MALDI-TOF MS), using a Microflex LT MALDI-TOF MS (Bruker Daltonics, Billerica, MA, USA). The agar plates were further incubated for an additional 72 h, and any new bacterial growth that was observed was analyzed with MALDI-TOF MS.

### Statistical analysis

The data analysis was performed in R version 4.3.0 using the stats, car, and performance packages (33–35). McNemar’s test for paired categorical data was used to assess whether the number of fecal samples in which *C. jejuni* and *C. hyointestinalis* could be detected differed significantly between early and late summer (sampling occasions 1 and 4). Univariate Pearson’s *χ*^2^ test was used to assess whether the choice of broth or selective media has an association with the detection of *Campylobacter*. The combined relationship between broth, selective media, and the detection of the bacteria was modeled using logistic generalized linear mixed models (GLMM), where the hierarchical structure of repeated samples for the same animal was modeled as a random effect. Both additive and biologically plausible interaction models were considered. These models were defined as


(1) $$\begin{array}{c} logit\left(P\left(Y=1\right)\right)=\;\beta_{0}+\;\beta_{1}\cdot broth+\;\beta_{2}\;\cdot selective\;media+u_{animal}+v_{animal:time} \end{array}$$

(2) $$\begin{array}{rl} \text{logit}(P(Y=1))= & \beta_{0}+\beta_{1}\cdot \text{broth}+\beta_{2}\cdot \text{selective media}\\ & +\beta_{3}\cdot (\text{broth}\cdot \text{selective media})+u_{\text{animal}}+v_{\text{animal:time}} \end{array}$$

The model goodness of fit was evaluated using the likelihood ratio test and the Akaike information criterion (AIC) to compare the models’ relative performance. Based on these evaluations, the additive model was chosen for *C. jejuni* due to negligible improvement in fit and non-significant interaction terms. In contrast, the interaction model was selected for *C. hyointestinalis* because it showed a significantly lower AIC and a significant likelihood ratio test for the interaction term. For model results, the fixed-effect coefficients and their 95% confidence intervals were extracted from the model summaries, while the *P* values for model terms were obtained from Type II Wald *χ*^2^ tests using the analysis of variance function from the car package. To visualize the interaction in the *C. hyointestinalis* model, predicted probabilities of bacterial detection were derived from the fitted GLMM for broth and selective media combinations and presented in an interaction effect plot.

## RESULTS

On all sampling occasions, *Campylobacter jejuni* and *Campylobacter hyointestinalis* were the only detected *Campylobacter* spp. (Table 2). Four cows (cows 3, 11, 15, and 17) remained negative for *C. jejuni* at all sampling occasions, whereas all cows tested positive for *C. hyointestinalis* on at least one occasion. A trend was observed with higher occurrence of *C. jejuni* at the beginning of the summer and higher occurrence of *C. hyointestinalis* at the end of the summer. However, McNemar’s test showed that the higher number of cows colonized with *C. jejuni* in sampling occasion 1 compared to occasion 4 was not statistically significant (*χ*² =3.2, df = 1, *P* = 0.07). Similarly, there was no significant difference in the number of cows colonized with *C. hyointestinalis* between the two occasions (*χ*² =0.8, df = 1, *P* = 0.37). A slight change was also seen in the number of cows simultaneously colonized with both *C. jejuni* and *C. hyointestinalis*, but this was not statistically significant (*χ*² =0.00, df = 1, *P* = 1.00).

**TABLE 2:** Summary of the number of cows colonized with *Campylobacter jejuni* and/or *Campylobacter hyointestinalis* at each sampling occasion as determined by any of the detection methods

Sampling occasion	No. of animals	*C. jejuni*		*C. hyointestinalis*		*C. jejuni* and *C. hyointestinalis*	
		Positive (*n*)	Prevalence (%)	Positive (*n*)	Prevalence (%)	Positive (*n*)	Prevalence (%)
1	18	12	66.7	14	77.8	8	44.4
2	18	11	61.1	17	94.4	9	50
3	18	8	44.4	16	88.9	7	38.9
4	17	7	41.2	16	94.1	7	41.2

Most *Campylobacter* isolates were detected after 48 h of incubation, and some additional isolates were observed following the extended 72 h incubation. In addition to *Campylobacter* spp., the most frequently identified non-*Campylobacter* spp. by MALDI-TOF MS were *Lactococcus lactis*, *Escherichia coli*, and *Pichia kudriavzevii*. Their occurrence was not analyzed in relation to culture method.

**Fig 2:**
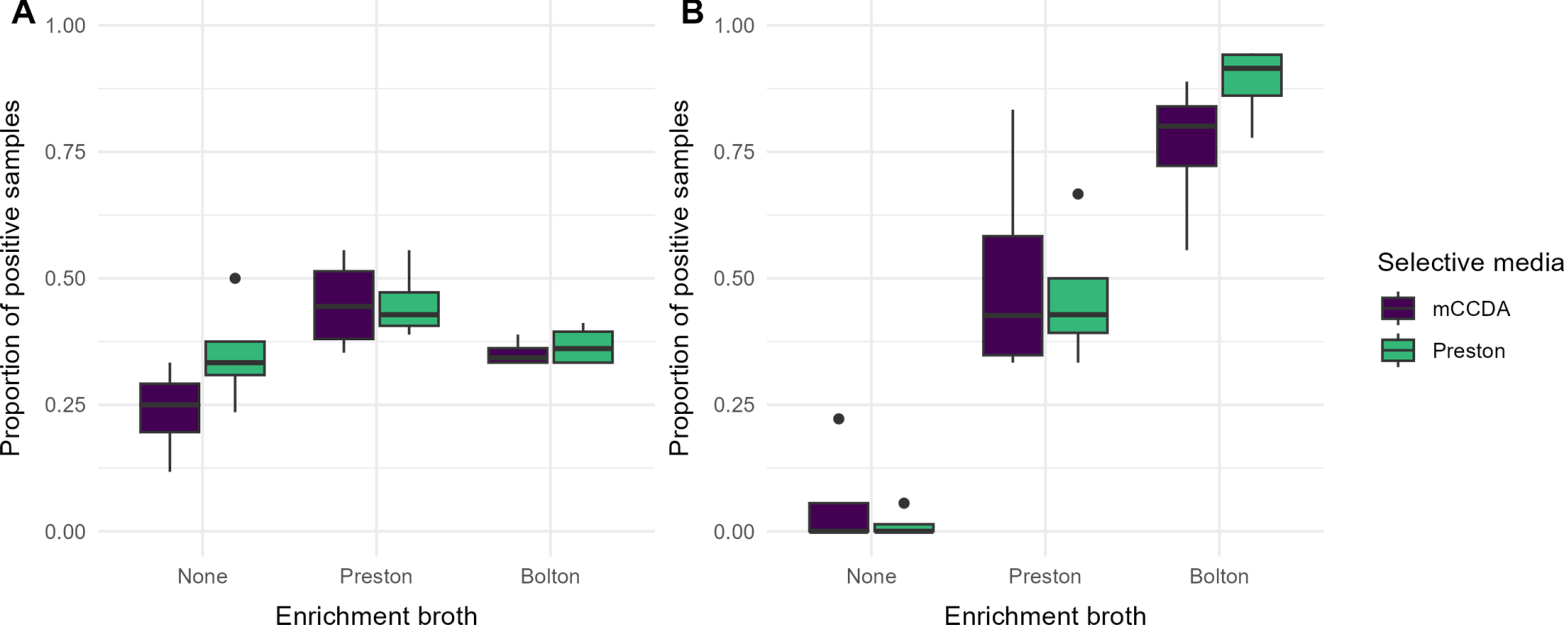
Detection of *C. jejuni* (**A**) and *C. hyointestinalis* (**B**) using different enrichment and selective agar combinations. Boxplots summarize proportions of positive samples across four sampling time points. Dots indicate values classified as outliers by the boxplot’s interquartile range method.

The number of detected *C. jejuni* and *C. hyointestinalis* varied, depending on the methods used, and the methods showed different performances for each species. The data showed that *C. hyointestinalis* prefers enrichment in Bolton broth before cultivation on Preston agar. In contrast, *C. jejuni* preferred enrichment in Preston broth before cultivation on mCCDA or Preston agar. Furthermore, direct culture on mCCDA was more suitable for *C. jejuni* than for *C. hyointestinalis* (Fig. 2).

The univariate Pearson’s *χ*^2^ test revealed a statistically significant association between the type of enrichment broth and bacterial growth (Table 3). This association was less pronounced with *C. jejuni* (*P* = 0.025) compared to *C. hyointestinalis* (*P* < 0.001), which showed a preference for enrichment in Bolton broth. No significant association between growth on selective agar plates and bacterial species was observed.

**TABLE 3:** Association between enrichment broth and selective agar and the *C. jejuni* or *C. hyointestinalis* result based on Pearson’s *χ*^2^ test^*a*^

Media	*C. jejuni*				*C. hyointestinalis*			
	Negative (*N* = 269)	Positive (*N* = 157)	*χ* ^2^	*P* value	Negative (*N* = 235)	Positive (*N* = 191)	*χ* ^2^	*P* value
Broth^*b*^			7.4	0.025			180	<0.001
None	100 (37%)	42 (27%)			137 (58%)	5 (2.6%)		
Preston	78 (29%)	64 (41%)			73 (31%)	69 (36%)		
Bolton	91 (34%)	51 (32%)			25 (11%)	117 (61%)		
Selective^*c*^			0.65	0.4			0.04	0.8
mCCDA	139 (52%)	74 (47%)			119 (51%)	94 (49%)		
Preston	130 (48%)	83 (53%)			116 (49%)	97 (51%)		

^*a*^ *N*, the number of individual fecal samples tested (i.e., each unique cow × sampling occasion combination).

^*b*^ Broth denotes the type of enrichment broth used (none means direct culture without enrichment).

^*c*^ Selective denotes agar medium used (includes both enrichment and direct cultures).

**Fig 3:**
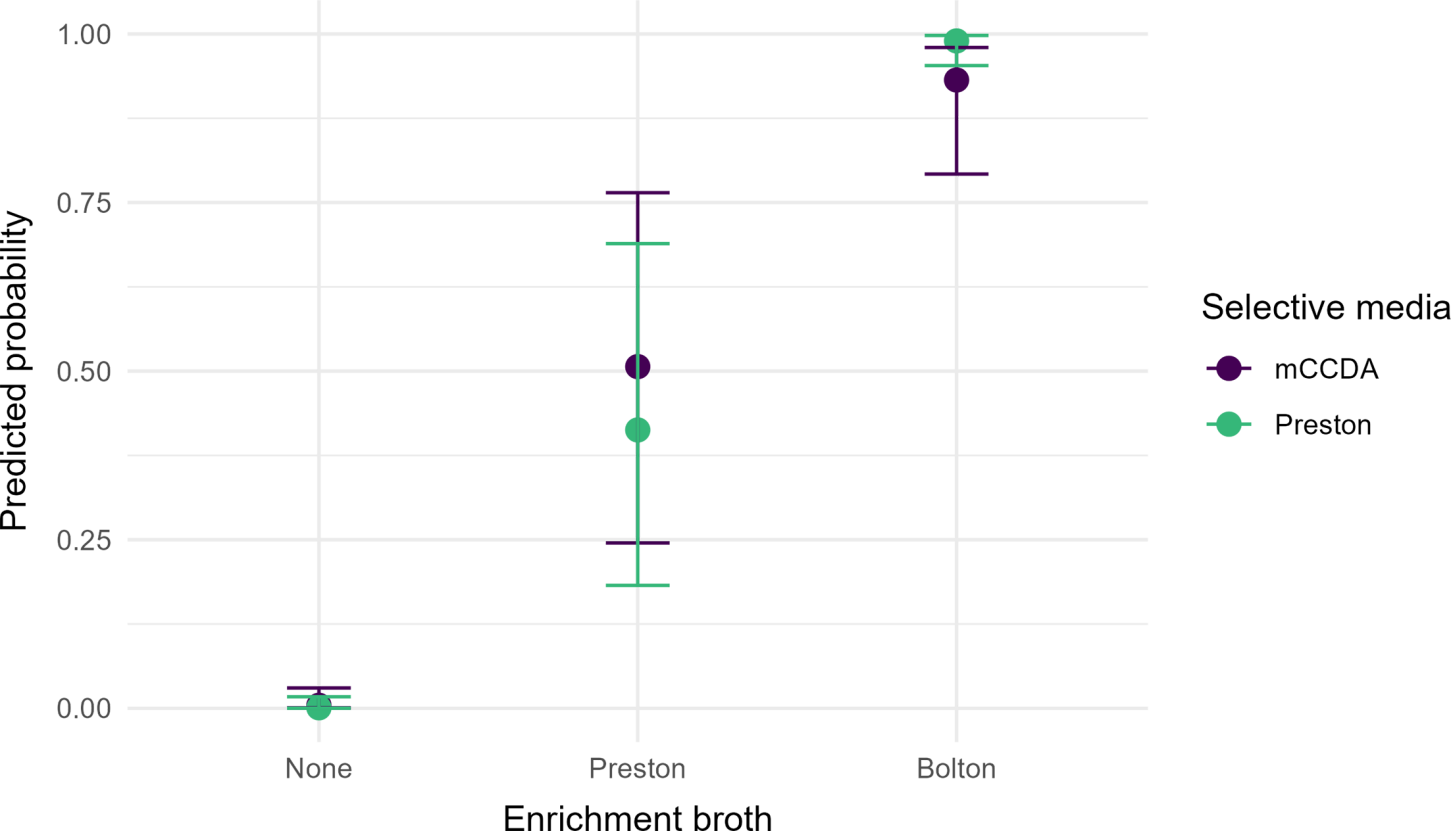
Interaction effect plot from the fitted *C. hyointestinalis* model, showing predicted probabilities of detection with corresponding 95% confidence intervals across different broth and selective media combinations.

The GLMM models showed a statistically significant association between the broth used and the detection of *C. jejuni and C. hyointestinalis* (Table 4). For clarity, model results are presented as odds ratios in the text and as log-odds coefficients in Table 4. Compared to the direct culturing, the odds ratio for detecting *C. jejuni* was 8.52 times higher (95% confidence interval [CI]: 3.16–22.92) for Preston broth and 2.41 times higher (95% CI: 0.99–5.88) for Bolton broth. The association was more pronounced with *C. hyointestinalis*, with odds 231 times higher (95% CI: 34–1,556) for enrichment with Preston broth and 3,075 times higher (95% CI: 273–34,651) for enrichment with Bolton broth. No significant association was observed between the selective agar media and the detection of the bacteria. A significant interaction between broth and selective agar was observed for *C. hyointestinalis* (*P* = 0.012), suggesting that the effect of direct culture or broth enrichment on bacterial detection varied, depending on the selective medium used. This interaction was further illustrated in an interaction effect plot (Fig. 3). The GLMM model residuals were independent and not autocorrelated, as the *P* values for the Durbin-Watson statistic for the *C. jejuni* and *C. hyointestinalis* models were 0.302 and 0.282, respectively.

**TABLE 4:** Logistic regression analysis of enrichment broth and selective agar media effects on detection of *C. jejuni* (additive model) and *C. hyointestinalis* (interaction model)^*a*^*^,^*^*b*^

Media^*c*^	*C. jejuni*			*C. hyointestinalis*		
	Coef	95% CI	*P* value	Coef	95% CI	*P* value
Broth			<0.001			<0.001
Preston	2.14	(1.15–3.13)		5.44	(3.54–7.35)	
Bolton	0.88	(−0.01 to 1.77)		8.03	(5.61–10.45)	
Selective			0.109			0.689
Preston	0.59	(−0.13 to 1.31)			−1.86	(−4.37 to 0.66)	
Broth × selective			–^*d*^			0.012
Preston × Preston	–^*d*^	–^*d*^		1.48	(−1.22 to 4.17)	
Bolton × Preston	–^*d*^	–^*d*^		3.81	(0.84–6.77)	

^*a*^ CI, confidence interval; Coef, coefficient (log-odds).

^*b*^ Coefficients and 95% CIs were obtained from model estimates, and *P* values were obtained from Type II analysis of variance.

^*c*^ Reference levels: broth denotes none (direct culture); selective denotes mCCDA.

^*d*^ Interaction terms were not included in the *C. jejuni* model.

## DISCUSSION

This study used different culture-based methods to compare their performance in detecting *Campylobacter* spp. in feces from cattle. While the overall prevalence of *C. jejuni* in the study herd was on the same level as commonly reported in adult animals in previous studies (20, 24), the mean proportion of cows in which *C. jejuni* was detected by direct culture (24% and 35% when using mCCDA and Preston agar, respectively) was considerably lower than in the previous study of Swedish cattle by Hansson et al. (20). This difference could be explained by temporal and between-herd variations, as well as differences in the concentration of bacteria in the feces. Additionally, the lubricant used in the rectal collection of fecal samples could potentially have inhibited the growth of the bacteria, as the exact ingredients of the lubricant were unknown.

The observed prevalence for *C. hyointestinalis* was substantially higher than what has been reported in previous studies (24, 25). Interestingly, no other *Campylobacter* spp. such as *C. coli* or *C. fetus* were found in this herd. However, this study was conducted only in a single herd, whereas other Swedish studies involving multiple herds yielded different results, isolating not only *C. jejuni* and *C. hyointestinalis* but also *C. lari*, *C. coli*, and *C. fetus* subsp. *fetus* (20, 36). It also cannot be excluded that some cows were carriers of other *Campylobacter* spp., which were not detected. While selecting colonies based on different macroscopic morphology could overlook different species with similar appearance and/or lower concentrations, the variety of methods used on the same sample should have increased the likelihood of detecting further species.

Regardless, the results imply that the prevalence of *C. hyointestinalis* in cattle may have been previously underestimated, which is likely due to their non-thermotolerant and fastidious nature (8, 29). While the association between incubation temperature and the detection of *C. hyointestinalis* was not studied, the temperature was likely a major factor impacting the detection of the bacteria. It should also be noted that the *Campylobacter* spp. were identified using MALDI-TOF MS, which has limitations in distinguishing *C. hyointestinalis* at the subspecies level. However, as *C. hyointestinalis* subsp. *hyointestinalis* is more commonly associated with cattle than the subspecies *lawsonii* (5), it is more likely that the isolates found in this study belonged to the subspecies *hyointestinalis*.

The results showed that the use and selection of enrichment broth had a statistically significant association with the detection of *C. jejuni* and *C. hyointestinalis*. This association was more pronounced for *C. hyointestinalis*, which could also be visually observed in the data (Fig. 2). These results highlighted that the best methods for detecting *Campylobacter* vary between the species. For detecting *C. hyointestinalis*, the direct culture performed poorly, whereas the odds for detection were significantly higher when selective culture was used. One possible explanation is that the concentration of *C. hyointestinalis* in the feces was generally low, making the amount of feces (<1 g) used for direct culture insufficient for reliable detection. For *C. jejuni*, the difference in performance between direct culture and enrichment was considerably lower than what was observed for *C. hyointestinalis*, likely due to the higher concentration of *C. jejuni* in the fecal samples. In general, when comparing the different method combinations, Bolton broth was the most likely to detect *C. hyointestinalis*, while Preston broth was the best method for detectin*g C. jejuni*.

Interestingly, assessing the goodness of fit for the GLMM models suggested different models for *C. jejuni* and *C. hyointestinalis*. Adding an interaction term between broth and selective media significantly improved the model fit only for *C. hyointestinalis*. This interaction was also statistically significant in the fitted model, indicating that the type of selective media used had a synergistic or conditional influence that better explained the detection variability. However, as visualized in the interaction effect plot, neither selective media consistently yielded higher detection probabilities, suggesting that the effectiveness of selective media was influenced by the prior broth enrichment conditions.

Notably, the odds ratio for detecting *C. hyointestinalis* with Bolton broth enrichment compared to direct culture was extremely high in the GLMM model (odds ratio = 3,075.28; 95% CI: 272.93–34,650.6). This large effect size reflects the strong improvement in detection associated with broth enrichment. However, a crude calculation based on the raw counts from Table 3 yields an unadjusted odds ratio of approximately 128.2 (95% CI: 47.5–345.7). The higher model-based estimate likely reflects the adjustment for repeated measures within animals and accounts for within-cow correlations, which the simple calculation does not consider. In addition, the near-complete lack of *C. hyointestinalis* detection by direct culture (only five positive samples) versus very high detection rates after Bolton enrichment (117 positives) can amplify the estimated odds ratio in the modeling framework. Such high odds ratios are plausible in microbiological studies where detection sensitivity between methods differs dramatically, but they should be interpreted cautiously given the wide confidence intervals observed both in crude and model-based analyses.

In summary, this study demonstrated that the choice of enrichment and culture methods substantially influenced the detection of *Campylobacter* spp. in dairy cattle fecal samples. The findings highlighted that *C. hyointestinalis* may have been more prevalent than previously assumed, potentially due to limitations in culturing protocols. Based on the results, we conclude that enrichment in Bolton broth followed by cultivation on Preston agar was the best method combination for detecting *C. hyointestinalis*, while *C. jejuni* was most consistently detected using Preston broth with either mCCDA or Preston agar. However, if only a single isolation protocol can be used, enrichment in Bolton broth (microaerophilic, with incubation at 37°C) followed by plating on Preston agar appears to be an effective method for recovering both thermotolerant *C. jejuni* and non-thermotolerant *C. hyointestinalis*. While the standards support using higher incubation temperatures for *C. jejuni*, in this study, Bolton broth and Preston agar combination still yielded the majority of *C. jejuni* isolates in our samples and had the advantage of detecting *C. hyointestinalis*, which would have been missed by the thermotolerant-only approach. These results underscore the importance of adapting detection methods when analyzing samples for non-thermotolerant *Campylobacte*r spp.

## Data Availability

The data set generated and analyzed during the study is provided in CSV format in the Supplemental Material.

## SUPPLEMENTAL MATERIAL

Supplemental Material spectrum.01475-25-s0001.csv Results from the sample analysis that were used in the statistical analysis.

ASM does not own the copyrights to Supplemental Material that may be linked to, or accessed through, an article. The authors have granted ASM a non-exclusive, world-wide license to publish the Supplemental Material files. Please contact the corresponding author directly for reuse.
